# Non‐muscle myosin II regulates aortic stiffness through effects on specific focal adhesion proteins and the non‐muscle cortical cytoskeleton

**DOI:** 10.1111/jcmm.16170

**Published:** 2021-02-06

**Authors:** Kuldeep Singh, Anne B. Kim, Kathleen G. Morgan

**Affiliations:** ^1^ Department of Health Sciences Boston University Boston MA USA; ^2^ CSIR‐Institute of Himalayan Bioresource Technology Palampur India

**Keywords:** aortic stiffness, contractility, focal adhesion, non‐muscle myosin II, smooth muscle contraction

## Abstract

Non‐muscle myosin II (NMII) plays a role in many fundamental cellular processes including cell adhesion, migration, and cytokinesis. However, its role in mammalian vascular function is not well understood. Here, we investigated the function of NMII in the biomechanical and signalling properties of mouse aorta. We found that blebbistatin, an inhibitor of NMII, decreases agonist‐induced aortic stress and stiffness in a dose‐dependent manner. We also specifically demonstrate that in freshly isolated, contractile, aortic smooth muscle cells, the non‐muscle myosin IIA (NMIIA) isoform is associated with contractile filaments in the core of the cell as well as those in the non‐muscle cell cortex. However, the non‐muscle myosin IIB (NMIIB) isoform is excluded from the cell cortex and colocalizes only with contractile filaments. Furthermore, both siRNA knockdown of NMIIA and NMIIB isoforms in the differentiated A7r5 smooth muscle cell line and blebbistatin‐mediated inhibition of NM myosin II suppress agonist‐activated increases in phosphorylation of the focal adhesion proteins FAK Y925 and paxillin Y118. Thus, we show in the present study, for the first time that NMII regulates aortic stiffness and stress and that this regulation is mediated through the tension‐dependent phosphorylation of the focal adhesion proteins FAK and paxillin.

## 1 INTRODUCTION

Non‐muscle myosin II (NMII) is a member of the conventional class II myosin family. Contrary to its name, NMII is expressed in essentially all eukaryotic cells including muscle cells and plays a role in many fundamental cellular and developmental processes such as cell adhesion,^1^, ^2^ cell migration,^3^ and cytokinesis.^4^ Similar to smooth muscle myosin II, NMII is composed of two heavy chains (230 kD), two regulatory light chains (20 kD), and two essential light chains (17 kD).^5^ Enzymatic activity is known to be regulated by phosphorylation of both regulatory light chains and heavy chains.^6^

There are three NMII isoforms: non‐muscle myosin IIA (NMIIA), non‐muscle myosin IIB (NMIIB) and non‐muscle myosin IIC (NMIIC). The corresponding heavy chains are encoded by three different genes (*MYH9*, *MYH10* and *MYH14*, respectively), which are located on three different chromosomes.^7^, ^8^ Although all three NMII isoforms contain similar domain structures and exhibit 60%‐80% amino acid sequence identity,^9^ they have different expressions and intracellular localizations in different cell and tissue types. NMIIA and NMIIB isoforms are expressed at a high level in many smooth muscle tissues, whereas NMIIC expression is low in SM tissue and high in neuronal tissue.^7^ Ablation of NMIIA and NMIIB in the whole mouse results in lethality at an embryonic stage,^2^, ^10^, ^11^ whereas ablation of NMIIC causes no known phenotypic change.^12^ In addition to the classical roles in cell adhesion, cell migration, and cytokinesis, a few studies have reported NMII involvement in the regulation of the biomechanics of smooth muscle contraction.^6^, ^13^, ^14^, ^15^ However, the subcellular molecular mechanisms by which NMII regulate smooth muscle biomechanics remain unclear and are likely to be tissue‐specific.

Stiffness is an important biomechanical property of the aorta. Aortic stiffness increases with age and is an independent predictor of negative cardiovascular outcome, including hypertension, stroke, kidney disease and vascular dementia.^16^, ^17^, ^18^, ^19^ Recent studies have shown that vascular smooth muscle cells (VSMCs) contribute significantly to the total vascular wall stiffness.^20^, ^21^ There are three dynamic components of the VSM cell that have been shown to contribute to VSMC stiffness: first, the cyclic attachment of cross‐bridges in contractile filament; second, the remodelling and transmission of force and stiffness through a non‐muscle actin cytoskeleton^22^, ^23^; and third, remodelling of focal adhesion complexes connected to the non‐muscle actin cytoskeleton.^24^ Although NMII has been shown to be involved in the cell‐mediated extracellular matrix reorganization and the resulting changes to the matrix stiffness,^25^ its possible role in the regulation of VSM cell stiffness has not been investigated.

The goal of this study was to determine whether NMII plays a role in the biomechanical properties of the aortic wall, and if so, to determine the molecular mechanisms. Here we show that NMII is, indeed, involved in the regulation of both aortic stress and stiffness. Furthermore, we show that the molecular mechanisms by which NMII regulates aortic stiffness include tension‐dependent alterations of the phosphorylation state of smooth muscle focal adhesion proteins and the cortical non‐muscle actin cytoskeleton of the smooth muscle cell.

## 2 MATERIALS AND METHODS

### 2.1 Animals and aortic tissue preparation

C57BL/6J adult male mice (~3‐4 months old) were used in this study. All the procedures were performed according to a protocol approved by the Boston University IACUC. Mice were killed by inhalation with an overdose of isoflurane in a closed chamber. Aortic tissue was quickly excised, rinsed and placed in an ice‐cold oxygenated (95% O_2_‐5% CO_2_) physiological saline solution (PSS; in mmol/L: 120 NaCl, 5.9 KCl, 11.5 Dextrose, 25 NaHCO_3_, 1.2 NaH_2_PO_4_, 1.2 MgCl_2_, 2.5 CaCl_2_; pH, 7.4). Axial rings (5 mm length) were cut from the thoracic aorta for biomechanics. At the end of the protocol, rings were quick‐frozen in a dry‐ice, acetone, 10 mmol/L dithiothreitol, 10% TCA slurry and stored at −80°C.

### 2.2 Measurement of aortic geometry and biomechanics

Axial length, diameter, and wall thickness of aortic tissue were quantitated to determine the cross‐sectional area and used to calculate aortic stress and stiffness. Ex vivo aortic stress and stiffness were measured as previously described.^20^, ^26^, ^27^ Briefly, thin triangular pieces of wire (0.01‐inch diameter) were threaded through the lumen of the aortic ring and suspended in an organ bath (50 ml) containing 37°C oxygenated PSS (95% O_2_‐5% CO_2_). The upper triangle was connected to a computer‐controlled motorized lever arm (Dual‐Mode Lever Arm System, Model 300C, Aurora Scientific), which also functions as a force transducer. Aortic rings were stretched in the circumferential direction to the previously determined optimum length (1.8 × slack length).

Aortic stiffness was determined by high‐frequency (40 Hz), small‐amplitude (1%) sinusoidal length perturbations. Aortic tissue stiffness is defined as the change in stress divided by the change in strain in response to the sinusoidal oscillations, (Δ*F*/*A*)/Δ*L*/*L*_0_) where Δ*F* is the amplitude of force output during cyclic stretches, *A* is the cross‐sectional area, Δ*L* is the amplitude of cyclic length changes, and *L*_0_ is the optimal length. Stress was calculated as force Δ*F* divided by the cross‐sectional area (*A* = 2*h*ι where *h* is the wall thickness and ι is the axial length of the ring).

### 2.3 Isolation of single differentiated smooth muscle cells from mouse aorta

To avoid any cytoskeletal changes that could occur during cell culture, freshly dissociated VSMCs (dVSMCs) were used in this study. Contractile dVSMCs were enzymatically isolated from mouse aorta tissue according to a previously published method^22^ and poured over glass coverslips on ice and allowed to adhere for 1 hour under an atmosphere of 100% oxygen.

### 2.4 Cell staining, immunofluorescence microscopy and colocalization quantification

dVSMCs were fixed with 4% paraformaldehyde for 20 minutes and washed twice with 0.1 mmol/L glycine in 1% BSA Hank's Balanced Salt Solution (HBSS), permeabilized with 0.1% Triton X‐100 for 10 minutes and blocked with 10% goat serum in 0.05% Triton X‐100 and 1% BSA HBSS for 30 minutes. Cells were incubated overnight with the desired primary antibody in 2% goat serum and 1% BSA HBSS. Cells were incubated with secondary antibody and mounted with fluoroshield with DAPI (Abcam). For γ‐cytoplasmic actin staining, cells were fixed with 1% paraformaldehyde for 20 minutes followed by ice‐cold methanol for 3 minutes. Cells were washed twice with HBSS containing 0.075% BSA.

For high‐resolution deconvolution microscopy, three‐dimensional image stacks were acquired with Nikon Eclipse TE 2000‐E inverted microscope using a Nikon Plan Apochromat 60X (Numerical Aperture 1.4) oil immersion objective and a charge‐coupled device camera (Cool SNAP HQ2, Photometrics). NIS‐Element Advanced Research software was used to capture images, and out‐of‐focus fluorescence was removed by deconvolution of *z*‐stacks. The degree of colocalization was quantified from merged images of different actin isoforms with NM II isoforms by using Pearson's correlation coefficient (PCC). The ‘colocalization tool’ in the NIKON NIS‐Element Advanced Research software was used to measure PCC values. For accurate measurement of the PCC, cells were carefully outlined to avoid any unlabelled extracellular regions that can artificially inflate PCC values if included in the region of measurement.^28^ A total of 20 cells (five cells from each group) were analysed for colocalization quantification.

### 2.5 A7r5 cell culture and siRNA transfection

A7r5 rat aortic SMCs (ATCC) were cultured in growth medium composed of Dulbecco's modified Eagle's medium (DMEM), supplemented with 10% foetal bovine serum and 1% glutamine and maintained under humidified conditions at 37°C and 5% CO_2_. Expression of NMII isoforms was inhibited by ON‐TARGET*plus* SMARTpool siRNAs specific for NMIIA and NMIIB, purchased from Horizon discovery. ON‐TARGETplus Non‐targeting Control siRNA was used as a control. Cells were seeded at a density of 1 × 10^5^ cells/mL and were transfected using DharmaFECT (Horizon Discovery), at a final concentration of 25 nmol/L in an antibiotic‐free Opti‐MEM medium according to the manufacturer's instructions. siRNA knockdown was analysed after 72 hours of transfection by Western blot using antibodies specific to NMIIA and NMIIB. Cells were lysed on ice, and samples were stored at −80°C.

### 2.6 Western blotting

Frozen aortic tissue was quickly homogenized and total protein measured as described previously by our group.^29^ Bands were normalized to GAPDH.

### 2.7 Actin polymerization assay

An actin polymerization assay was performed according to the protocol described previously.^30^ Each aortic strip was placed in a 0.5 mL tissue homogenization tube (Precellys, Bertin Technologies) containing pre‐warmed lysis and F‐actin stabilization buffer (in mmol/L: 50 PIPES pH 6.9, 50 NaCl, 5 MgCl_2_, 5 EGTA) 5% [V/V] Glycerol, 0.1% Nonidet P40, 0.1% Triton X‐100, 0.0% Tween 20, 0.1% 2‐mercapto‐ethanol, 0.001% Antifoam C with 1 mmol/L ATP and 1× protease inhibitor cocktail (Cytoskeleton, Inc). The tissue was quickly homogenized. 100 μL of homogenates was incubated at 37°C for 20 minutes and spun at 150 000 *g* for 60 minutes at 37°C in Optima TLX ultracentrifuge (Beckman Coulter). The supernatant (G‐actin) was carefully pipetted, 100 μL of ice‐cold F‐actin depolymerization buffer (8 mol/L urea) was added to each pellet, and the pellet was dissolved by triturating every 15 minutes for 1 hour. The solution was spun at 2300 *g* for 5 minutes at 4°C, and the supernatant collected to give the final F‐actin fraction. Equal volume of G and F‐actin samples was resolved by SDS‐PAGE and immunoblotting with anti‐actin primary antibody (Cytoskeleton).

### 2.8 Chemicals and antibodies

(−)‐Blebbistatin, primaquine and phenylephrine were purchased from Sigma. siRNA transfection reagents were purchased from Horizon Discovery. DPBA was purchased from Santa Cruz Biotechnology. The following primary antibodies were used: NMIIA (rabbit, 1:200), NMIIB (rabbit, 1:200), GAPDH (rabbit, 1:20 000) and α‐smooth muscle actin (mouse 1:10 000) from Sigma; β‐Cytoplasmic actin (mouse 1:500) and γ‐Cytoplasmic actin (mouse 1:500), a gift from Christine Chaponnier; phospho‐FAK Y925 (rabbit, 1:200) and phospho‐Paxillin Y118 (rabbit, 1:250) from Cell Signaling Technologies; FAK monoclonal antibody (mouse, 1:250) from Thermo Fisher Scientific; anti‐Paxillin (mouse, 1:500) from BD biosciences; anti‐actin antibody (rabbit, 1:500) from Cytoskeleton. For immunofluorescence experiments, goat anti‐mouse and goat anti‐rabbit Alexa Fluor 488 and Alexa Fluor 568 (1:1000, Invitrogen) were used as secondary antibodies. For Western blotting experiments, IRDye 680 RD and IRDye 800CW labelled goat anti‐rabbit and goat anti‐mouse (LI‐COR, 1:10 000) were used as secondary antibodies.

### 2.9 Statistical analysis

All results are presented as mean ± SEM Analysis was carried out using the GraphPad Prism 8 software. Two‐tailed student's *t* tests were used when comparing values between two experimental groups. The significance of differences between two individual datasets was taken at *P* < .05. We used two‐way ANOVA to compare parameters among treatment groups followed by Turkey's multiple comparisons test.

## 3 RESULTS

### 3.1 Ex vivo inhibition of non‐muscle myosin II in the mouse inhibits alpha agonist‐activated aortic stress and stiffness

To elucidate the role of NMII in vascular function, we used a small molecule inhibitor of NMII, blebbistatin (Bleb). Blebbistatin binds to the myosin ADP‐Pi complex of the myosin ATPase cycle and interferes with the release of phosphate; therefore, it blocks the myosin II head in the actin‐detached state and prevents actin‐myosin interactions.^31^ Based on in vitro and ex vivo characterization, blebbistatin has been shown to be a cell permeable selective inhibitor of NMII (IC_50_ ~ 0.5‐5 μmol/L) and has a low binding affinity towards smooth muscle myosin (SMII) (IC_50_ ~ 15‐80 μmol/L).^4^, ^31^, ^32^, ^33^, ^34^, ^35^, ^36^, ^37^, ^38^ Only the active (−)‐enantiomer of blebbistatin is known to inhibit NMII activity, and only this enantiomer was used in the present study.^32^, ^33^, ^39^

**Figure 1:**
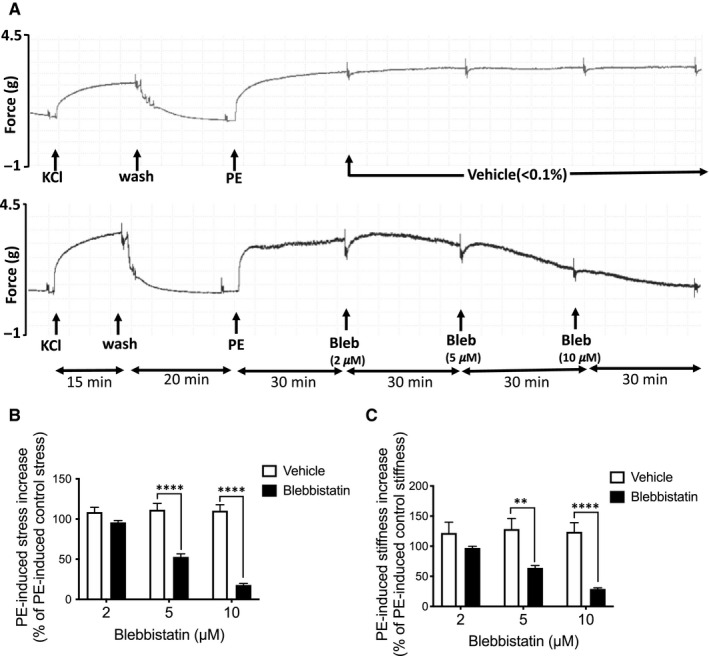
Effect of blebbistatin on biomechanical properties of aortic tissue activated by an alpha agonist. A, Isometric force development in control vehicle‐treated aorta tissue (upper panel) and blebbistatin‐treated aorta tissue (lower panel). Aortic rings were treated for 30 min with 2, 5 and 10 μmol/L blebbistatin after PE stimulation. B, Blebbistatin significantly inhibited PE‐induced aortic stress at 5 and 10μmol/L as compared to control vehicle‐treated tissues (n = 8). C, Blebbistatin significantly inhibited PE‐induced aortic stiffness at 5 and 10 μmol/L as compared to only vehicle‐treated tissues. No statistically significant inhibition was found at 2 μmol/L on aortic stress and stiffness as compared to vehicle‐treated tissues (n = 8). Values in graphs are normalized to PE‐induced control stress/stiffness. Control stress/stiffness refers to the stress/stiffness measured in the presence of PE before blebbistatin/vehicle treatment. ** and **** indicate *P* < 0.005 and <0.0001, respectively. Two‐way ANOVA

To determine the effective concentration of blebbistatin required to alter the biomechanics of mouse aorta tissue, we performed a dose response analysis. Cell viability was confirmed by the addition of 51 mmol/L KCl PSS to the tissue bath (Figure 1A). After returning to normal PSS, aortic tissues were treated with 10 μmol/L of the alpha agonist, phenylephrine. Once the phenylephrine‐induced contraction reached steady state (~30 minutes), either vehicle alone (DMSO) or incremental blebbistatin concentrations (2, 5 and 10 μmol/L) were added to the tissue bath. Stress and stiffness were measured at steady state. Dose response analysis revealed that blebbistatin significantly inhibits phenylephrine‐induced aortic stress at 5 and 10 μmol/L compared to control vehicle‐treated tissues (Figure 1B). The 2 μmol/L blebbistatin concentration had no statistically significant effect.

To investigate the role of NMII specifically in aortic stiffness, we used biomechanical techniques to quantitate agonist‐induced aortic stiffness in the presence and absence of blebbistatin.^30^ As seen in Figure 1C, blebbistatin significantly decreases phenylephrine‐induced increases in aortic stiffness at 5 and 10 μmol/L concentrations (n = 8) as compared to control tissues treated with vehicle only. However, similar to the aortic stress, no statistically significant effect was observed on aortic stiffness at 2 μmol/L concentration of blebbistatin as compared to control tissue.

### 3.2 Blebbistatin also decreases depolarization‐induced increases in aortic stress and stiffness

Multiple signalling pathways coexist in SM cells, which contribute to SM contractility and stiffness.^40^ Membrane depolarization activates SM by a signalling mechanism involving the activation of voltage gated Ca^2+^ channels, Ca^2+^ influx, Ca^2+^ binding to calmodulin, Ca^2+^‐calmodulin complex to activate myosin light chain kinase, phosphorylation of myosin light chains, and actin‐myosin cross‐bridge cycling and contraction.

**Figure 2:**
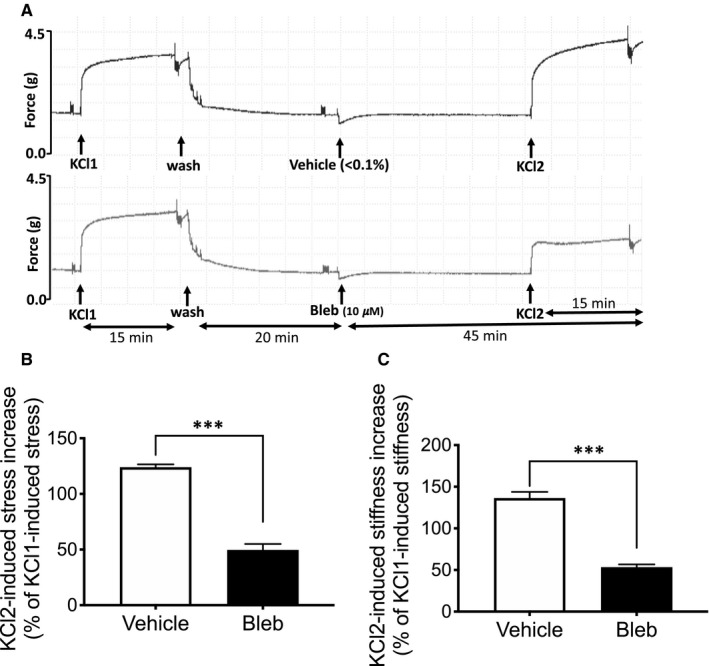
Blebbistatin inhibits depolarization‐induced aortic stress and stiffness. A, Typical time course of isometric force responses during depolarization of aortic tissue with 51 mmol/L KCl PSS in control tissue (upper panel) and tissue pre‐treated with blebbistatin for 30 min (lower panel). B, Blebbistatin significantly decreases depolarization‐induced stress in blebbistatin‐treated tissues (Vehicle control: 124 ± 2.5%, bleb: 49.6 ± 5.3%; n = 3). C, Inhibition of depolarization‐induced increase in aortic stiffness in blebbistatin pre‐treated tissue as compared to control vehicle‐treated tissues (Vehicle control: 136.3 ± 7.4%, bleb: 53.33 ± 3.3%; n = 3). The values are expressed as a % of KCl1‐induced increase in aortic stress and stiffness (average ± SEM). Two‐tailed unpaired *t* test ***P < .001

To determine whether NMII is involved in the depolarization‐mediated pathway of contraction, aorta tissues were treated with 10 μmol/L blebbistatin for 30 minutes followed by treatment with a depolarizing physiological saline solution containing 51 mmol/L KCl. Blebbistatin significantly decreases the KCl‐induced increase in aortic stress as compared to only vehicle‐treated tissues (Figure 2A,B). KCl‐induced aortic stiffness was also significantly decreased by blebbistatin (Figure 2C). These results indicate that NMII affects parts of signalling pathways common to both KCl and PE activation, either in the contractile filaments, focal adhesions or non‐muscle actin cytoskeleton.^40^

### 3.3 Isoform‐specific localization of actin isoforms and NMII isoforms in dVSMCs of mouse aorta

**Figure 3:**
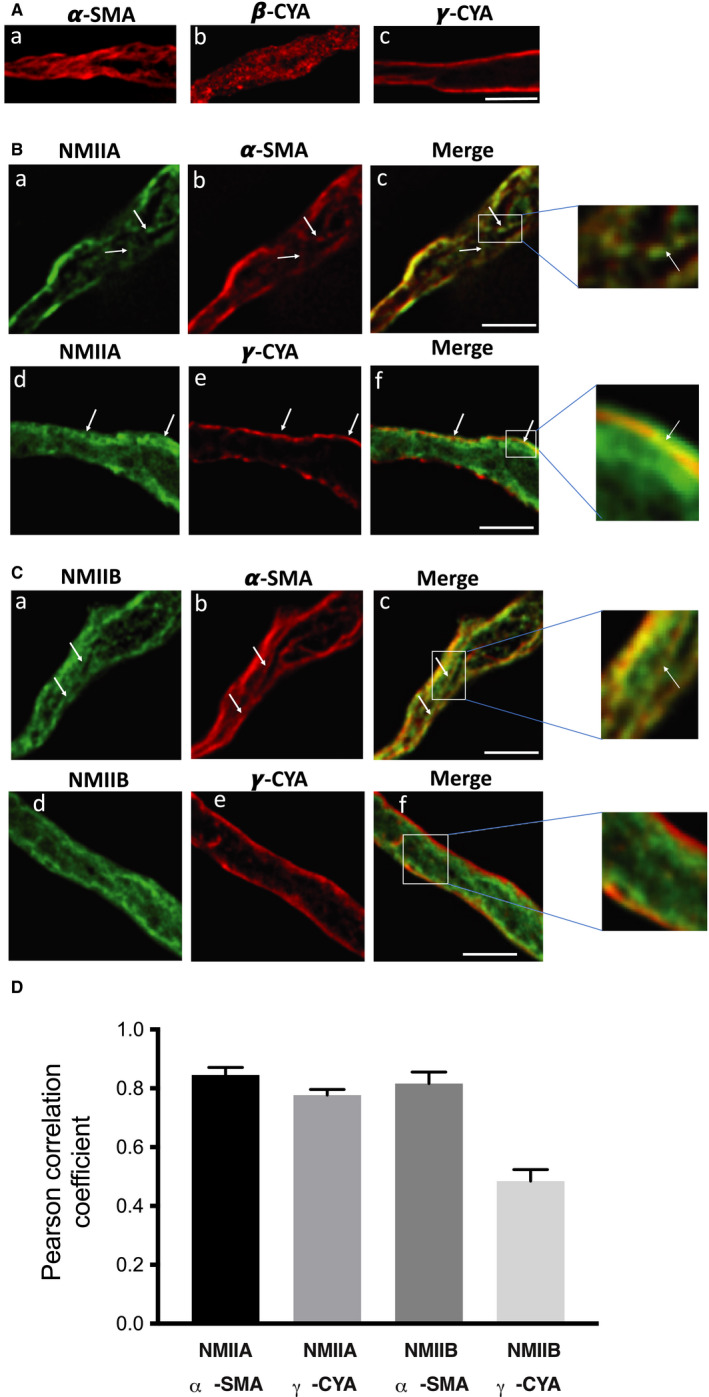
Subcellular localization of actin and NM myosin II isoforms in vascular smooth muscle cells of mouse aorta. A, Freshly enzymatically isolated dVSMCs from mouse aortas were fixed and probed with isoform‐specific antibodies for smooth muscle actin isoforms. Deconvolution immunofluorescence microscopy was used to increase the resolution of the imaging. Results indicate that α‐SMA (a) is present in central filaments, presumably the previously described contractile filaments.^43^ β‐CYA (b) is localized in punctate structures throughout the cell, and γ‐CYA (c) specifically is localized to the cell cortex. B, Colabeling was detected (Panel c) (arrows) of NMIIA (Panel a) with α‐SMA (Panel b) (upper row). Colabeling was detected (Panel f) (arrows) of NMIIA (Panel d) and γ‐CYA (Panel e) (lower row). Colocalization is detected digitally and displayed in merged images by yellow staining (c and f and at higher magnification to the right). C, Colocalization of NMIIB (a) with α‐SMA (b) in dVSMCs (upper row). Colocalization is detected digitally and shown in the merged image by yellow staining (c, enlarged view). NMIIB did not colocalize with γ‐CYA (lower row). A merged image is displayed in f (higher magnification to the right) and shows a lack of yellow staining. D, Summary graph of NM myosin II and actin isoform colocalization through the use of Pearson's correlation coefficients (PCC). PCC values are expressed as the mean ± SEM. Scale bar, 5 μm

NMII isoforms are reported to have distinct intracellular localization patterns in different cell types.^7^, ^41^, ^42^ The above results could be explained by an effect of NM II to regulate aortic stress and stiffness by interacting with (a) the contractile filaments, (b) the focal adhesions or (c) the NM actin cytoskeleton (see more details in Section 3.4). To study whether NMII localizes with the contractile filaments in the core of the cell or with the non‐muscle cytoskeleton adjacent to the plasmalemma, we enzymatically dissociated single dVSMCs from the mouse aorta and imaged the cells with high‐resolution deconvolution microscopy as previously described.^22^ Our laboratory has published that α‐smooth muscle actin associates with contractile filaments in the core of the VSMC, whereas γ‐cytoplasmic actin associates with the sub‐plasmalemmal cell cortex and β‐cytoplasmic actin is located in punctate dense bodies in ferret VSMC.^43^ Also, Dugina et al^44^ reported similar findings for actin isoforms in fibroblast and epithelial cells. To determine the localization of these actin isoforms in mouse aortic cells, we used isoform‐specific actin antibodies to localize different actin populations in mouse aortic smcs and found a similar pattern. α‐smooth muscle actin filaments (α‐SMA) appear to snake through the core of the cell (Figure 3Aa), but in contrast, β‐cytoplasmic actin (β‐CYA) is distributed throughout the cells in punctatae (Figure 3Ab). γ‐cytoplasmic actin (γ‐CYA), on the other hand, is confined to the cell cortex (Figure 3Ac).

We then costained for the NMII isoforms to allow comparison with the specific actin isoforms in the aortic smcs. We found that NMIIA colocalizes with α‐SMA filaments (Figure 3B, upper row), presumably in the contractile filaments in the core of the cell (Figure 3B upper row, enlarged view). Then, we costained for NMIIA and the cortical γ‐CYA (Figure 3B, lower row). The merged image shows that NMIIA also colocalizes with cortical γ‐CYA (Figure 3B, lower row, enlarged view).

Next, we investigated the localization of the NMIIB isoform (Figure 3C). First, we compared it with that of α‐SMA in the contractile filaments (Figure 3C, upper row). Similar to NMIIA, the merged image shows that NMIIB colocalizes with contractile filaments (Figure 3C upper row, enlarged view). However, when we co‐labelled NMIIB with γ‐CYA (Figure 3C, lower row), there was a lack of yellow signal in the merged image, which indicates that NMIIB does not colocalize with cortical γ‐CYA (Figure 3C lower row, enlarged view). This difference in the localization of NMIIA and NMIIB in the cell cortex suggests that NMIIA has an additional function that NMIIB does not share in the function of cortical γ‐actin filaments, even though they may have similar functions in the contractile filaments.

To objectively quantify the degree of colocalization between NMII isoforms and actin isoforms, Pearson's correlation coefficients (PCC) were calculated using the ‘Colocalization’ tool in Nikon NIS‐Element Advanced Research software. PCC values near zero indicate a lack of correlation between fluorescence intensities of two images, whereas a PCC value of 1 indicates a perfect linear relationship between the two images. A high degree of colocalization was indicated in merged images of NMIIA/α‐SMA, NMIIA/γ‐CYA and NMIIB/α‐SMA because the PCC values were 0.84 ± 0.02, 0.77 ± 0.01 and 0.81 ± 0.03, respectively. In contrast, a relatively low degree of colocalization (PCC = 0.48 ± 0.03) was indicated for the NMIIB/γ‐CYA images (Figure 3D). A total of 20 cells (five from each group) were analysed for quantification.

### 3.4 Non‐muscle myosin II regulates aortic stiffness via inhibition of tyrosine phosphorylation of focal adhesion proteins

Focal adhesions (FA) are protein complexes near the cell membrane that connect the actin cytoskeleton to the extracellular matrix via integrins in many different cell types.^27^, ^45^ Agonist stimulation of VSMCs activates a signalling cascade that ultimately increases actomyosin cross‐bridge cycling. The mechanical force generated by the actomyosin cross‐bridge cycling, in turn, increases the tension on the non‐muscle cytoskeleton and activates the engagement of FA proteins (focal adhesion kinase (FAK), talin and paxillin) with the integrins. It has previously been shown in several systems that, in non‐muscle cells, physical tension on the integrins leads to autophosphorylation of FAK at tyrosine 397, which then causes this site to bind to the Src homology domain (SH2) of the Src protein. It has been demonstrated for many cell types that the formation of this FAK‐Src complex promotes the subsequent phosphorylation of paxillin at Y118 and FAK at Y925, Y576, Y577 and Y861.^46^, ^47^ The stimulus‐induced increase in moderate tension at the focal adhesion in non‐muscle cells is known to lead to the growth of the FA^48^ and, in smooth muscle cells, leads to further increases in force and stiffness.^27^, ^30^

**Figure 4:**
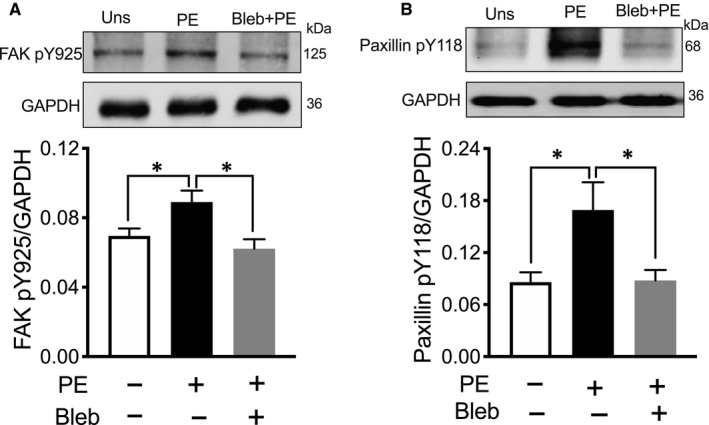
The NM myosin II inhibitor, Blebbistatin, suppresses phenylephrine‐induced phosphorylation of focal adhesion proteins. A, Representative FAK Tyr 925 phosphorylation immunoblots of tissue extracts from unstimulated preparations (Uns), PE‐stimulated preparations and PE‐stimulated preparations in the presence of 10 μmol/L blebbistatin (top). Mean results are shown in the bottom panel. FAK Tyr 925 phosphorylation increased significantly in response to 10^−5^ mol/L PE. Blebbistatin significantly inhibited the PE‐induced increase in FAK Tyr 925 phosphorylation (n = 6). B, Representative Paxillin Tyr 118 phosphorylation immunoblots of tissue extracts of unstimulated, PE‐stimulated and PE‐stimulated preparations in the presence of 10 μmol/L blebbistatin (top). Mean results (bottom) show that Paxillin Tyr 118 phosphorylation increases in response to PE in a blebbistatin‐inhibitable manner (n = 6). *P < 0.05, unpaired student *t* test

To determine whether NMII modulates aortic stiffness via effects on FA signalling in VSMCs, we compared the agonist‐induced phosphorylation of FAK and paxillin in the presence and absence of blebbistatin. FAK Y925 is a Src substrate that signals downstream to ERK and ultimately regulates the aortic stiffness and contractility. The alpha agonist phenylephrine (PE) increases the phosphorylation of FAK Y925 and paxillin Y118. Interestingly, the PE‐induced increase in phosphorylation of FAK and paxillin was significantly inhibited by blebbistatin (n = 6; Figure 4A,B). These data indicate that NMII is required for the stimulus‐induced and tension‐dependent phosphorylation of FA proteins. The data also point to a possible role for NMII in the tension‐dependent growth of focal adhesions in disorders of increased aortic stiffness.^27^

**Figure 5:**
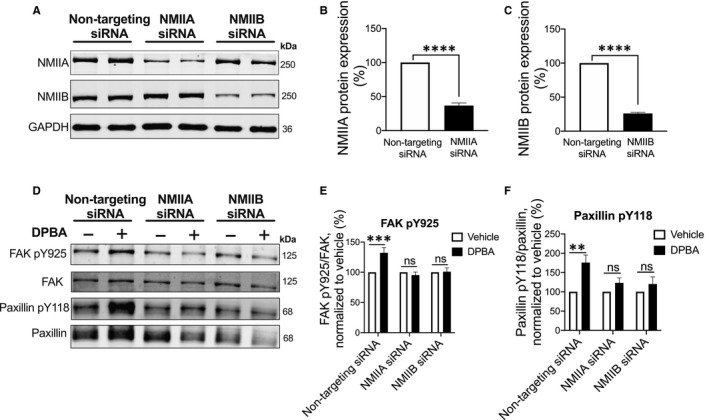
siRNA knockdown of NM myosin II isoforms in A7r5 cells down‐regulates agonist‐induced phosphorylation of focal adhesion proteins. A, A typical Western blot depicting isoform‐specific knockdown of NMIIA and NMIIB. B and C showing depletion in NMIIA (n = 6) and NMIIB (n = 9) protein levels after 72 h siRNA knockdown as compared to non‐targeting siRNA‐transfected control cells. Statistical significance was tested by two‐tailed unpaired *t* test, ****P < 0.0001. D, Representative immunoblot of DPBA‐induced phosphorylation of FAK and paxillin in siRNA‐transfected cells. E and F, Densitometry analysis of Western blots shows that DPBA‐induced increase in tyrosine phosphorylation of FAK Y925 and Paxillin Y118 is suppressed in both NMIIA and NMIIB siRNA‐transfected cells (n = 6). Statistical significance was tested by Two‐way ANOVA, Turkey's multiple comparison test, mean ± SEM **P < 0.005, ***P < 0.001, ns, non‐significant

Next, to validate the blebbistatin effect on phosphorylation of FA proteins, we used a complimentary siRNA knockdown approach to inhibit specific NMII isoforms in serum‐starved A7r5 cells, a differentiated SMC line from rat thoracic aorta.^49^ A7r5 cells were transfected with non‐targeting, and NMIIA‐ and NMIIB‐specific siRNAs in reduced serum media (OptiMEM). 72 hours post‐transfection, NMIIA and NMIIB protein expression was analysed by Western blot. Both NMIIA and NMIIB protein expression in siRNA‐transfected cells were reduced to ~65% and ~75%, respectively, as compared to non‐targeting siRNA‐transfected control cells (Figure 5A‐C). Further, to analyse the knockdown effect of NMII isoforms on FA signalling, 72 hours post‐transfected cells were stimulated with 3 μmol/L phorbol ester (DPBA) to quantitate the DPBA‐induced tyrosine phosphorylation of FA proteins FAK 925 and Paxillin 118. Similar to the above blebbistatin results, the DPBA‐induced increase in tyrosine phosphorylation of FAK 925 and Paxillin 118 was significantly suppressed in both NMIIA and NMIIB siRNA‐transfected cells as compared to non‐targeting control cells (Figure 5D‐F). Taken together, these findings indicate a role of NM myosin II in tension‐dependent phosphorylation of FA proteins which ultimately regulates aortic stiffness.

### 3.5 NM myosin II is not required for the endocytic recycling of focal adhesion proteins

**Figure 6:**
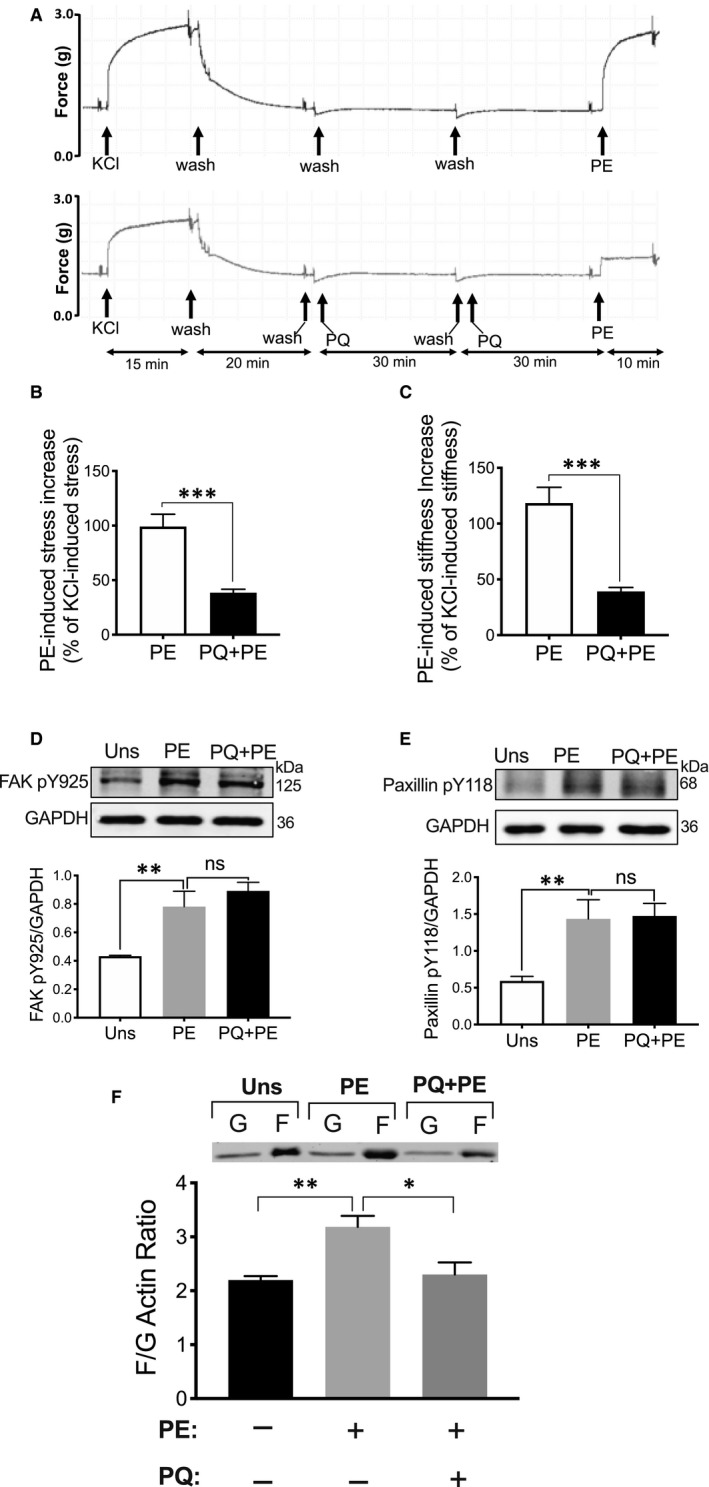
Effect of primaquine on aortic contractility and phosphorylation of focal adhesion proteins. A, Isometric force recording during stimulation of aortic tissue with phenylephrine in the absence (upper) and presence of primaquine (lower). B, Primaquine pre‐treatment significantly decreased PE‐induced aortic stress (n = 5). C, PE‐induced increase in aortic stiffness decreased in the presence of primaquine (n = 5). The values are presented as % of KCl‐induced contraction. D‐E, Primaquine has no effect on PE‐induced phosphorylation of FAK Y925 (n = 3) and paxillin Y118 (n = 6). Upper panels: typical Western blot images, Lower panel: mean densitometry analysis of raw images. F, Inhibition of PE‐induced actin polymerization by the primaquine (n = 5). *P < 0.05, **P < 0.005, ***P < 0.001, unpaired, two‐tailed Student's *t* test

FAs in contractile dVSM cells are dynamic in nature. Prior studies from our laboratory and those of others have shown that redistribution of FA proteins occurs^24^, ^50^, ^51^ in response to the addition of vasoconstrictors, and the cyclic remodelling of FA proteins requires an endocytic recycling pathway in vascular smooth muscles.^24^ Thus, we speculated that the endocytic recycling of the focal adhesion proteins might be NM myosin II‐dependent. To test this hypothesis, we first determined whether endosomes have any role in the regulation of aortic stiffness in VSM. We inhibited endosome function by treating the tissue for 1 hour with 150 μmol/L primaquine, an inhibitor of endosome budding. As seen in Figure 6A‐C, both PE‐induced aortic stress and stiffness decreased in the primaquine treated tissues as compared to control tissues.

Then, we asked whether endosomes are necessary for the regulation of phosphorylation of FA proteins observed here. Thus, we examined the phosphorylation of FAK and paxillin in the presence or absence of primaquine and found that PE increases the phosphorylation of FAK and paxillin at Y925 and Y118 sites, respectively; however, no significant changes were detectable in the phosphorylation of FAK and paxillin in the presence of primaquine (Figure 6D‐E). These data indicate that the endosomes are not necessary for the role of NMII in regulating FA phosphorylation and that the phosphorylation of FA proteins, which is regulated by NMII, must occur, temporally, before entry into the endosomes.

Additionally, the question arises as to how the endosome regulates aortic stiffness in VSM. Previous studies have shown that actin polymerization increases in the presence of agonists and that it is also a key regulator of vascular contractility and stiffness.^23^, ^30^ Thus, we tested whether endosomes regulate aortic stiffness via regulation of actin polymerization and whether actin polymerization is sensitive to primaquine treatment. Aortic tissues were treated in the presence and absence of primaquine. As shown by filamentous/globular actin ratios in Figure 6F, the PE‐induced increase in actin polymerization was significantly decreased in primaquine‐treated tissues. Thus, these data indicate that endosomes regulate aortic stiffness in VSMCs via an effect on actin polymerization and that this mechanism does not involve an effect on NMII.

## 4 DISCUSSION

The present study demonstrates that NMII regulates stress and stiffness in aortic SMC. Further, we show here that NMII not only localizes to the cell cortex and the contractile filaments, but also regulates aortic stress and stiffness via effects on FA signalling and the activity of the contractile filaments. Our data support the hypothesis that in addition to the SMII, NMII regulates tonic force maintenance and contributes to smooth muscle contractility as well as aortic stiffness, which therefore may provide a novel therapeutic target for the treatment of vascular disease.

These data support earlier findings from urinary bladder, in which mice deficient in smooth muscle myosin heavy chain (SM‐MHC) but expressing non‐muscle myosin heavy chain A and B (NM‐MHC A and B) can generate force and shortening in the smooth muscle.^14^ The fact that the bladder tissue of SMMHC‐deficient mice demonstrated a slowed tonic contraction and lacked an initial peak in force typical of bladder muscle also suggests that there are qualitative differences in the properties of the contractions caused by activity of NMII and SMII, respectively.^13^ Furthermore, urinary bladder tissue from newborn mice, which express high amounts of NMII, demonstrates an approximately 55% inhibition of force by blebbistatin, but adult bladder tissue, which expresses high amounts of SMII, is reported to be insensitive to blebbistatin.^52^ Taken together, these bladder studies and our current studies suggest a role of NMII in the regulation of smooth muscle contractility.

Of note, another group^39^ has reported an apparently low level of NMII in rabbit smooth muscles, but that blebbistatin concentration (~4‐5 μmol/L) inhibited the tonic contractions of adult rabbit femoral and saphenous arteries, suggesting either a significant functional effect of the low abundance NMII in this tissue or an action of blebbistatin separate from its inhibition of NMII. Therefore, the role of NMII in smooth muscle contraction may be organ‐specific and species‐specific.

In this study, we inhibited NMII with blebbistatin to determine its contribution in vascular contractility and stiffness in mouse aorta. Blebbistatin is known to selectively inhibit NMII (IC_50_ ~0.5‐5 μmol/L) and has a low affinity towards smooth muscle myosin II (IC_50_ ~80 μmol/L) as well as myosin classes I, V and X.^31^, ^32^, ^33^ However, one group has suggested that blebbistatin can also inhibit some smooth muscle myosin IIs (IC_50_ ~ 3‐6.5μmol/L).^39^, ^53^ The reasons for the discrepancy in IC_50_ values for SMII are not clear but may be because of the different preparations of smooth muscle myosin,^54^ species/tissue‐specific differences or protocol differences. In the present study, we show that blebbistatin significantly inhibits both PE‐induced stress and stiffness at 5 and 10 μmol/L (Figure 1A‐C) in mouse aorta. Our results are consistent with those of Rhee et al^55^ in which they showed that 15 and 25 μmol/L of active blebbistatin inhibits tonic force maintenance in both bladder and aorta tissue. Importantly, our results are first to show that blebbistatin inhibits agonist‐induced aortic stiffness which is a recognized risk factor associated with ageing‐induced vascular dementia, as well as kidney and heart disease.

Although NMII isoforms share considerable similarity in their amino acid sequence and molecular structure, they exhibit differential expression, function and subcellular localization in different tissues.^7^, ^37^, ^41^, ^42^ The NMIIA isoform is predominantly expressed in adult bladder tissue, whereas the NMIIB isoform is highly expressed in the aorta tissue.^13^, ^55^ Our results in freshly isolated dVSMCs from mouse aorta demonstrate that NMIIA isoforms colocalize with α‐SMA‐containing contractile filaments as well as with γ‐CYA at the cell cortex (Figure 3B). Moreover, we show that NMIIB colocalizes with α‐SMA contractile filaments. However, we did not observe colocalization of NMIIB with γ‐CYA a marker of cortical actin (Figure 3C).

**Figure 7:**
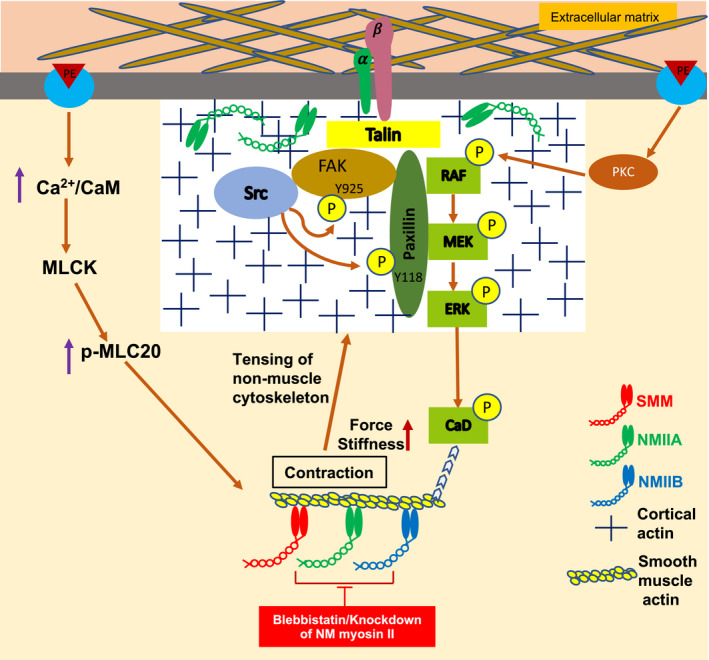
NM myosin II isoform function in the regulation of aortic stress and stiffness

NMMII isoforms have been implicated in actin and focal adhesion remodelling in many different cell types.^56^, ^57^, ^58^ A major finding of this study is that both blebbistatin and siRNA knockdown of NM myosin II isoforms suppress the agonist‐induced increase in tyrosine phosphorylation of FAK Y925 and paxillin Y118 (Figures 4 and 5). Previous studies have shown that multiple pathways coexist in SMC to regulate smooth muscle contraction (Figure 7). The alpha agonist phenylephrine has specifically been shown to activate multiple signalling pathways in VSM including (a) activation of calcium‐mediated signalling events which lead to activation of myosin light chain phosphorylation and actomyosin cross‐bridge cycling and contraction and (b) PKC activation leading to the activation of Raf, MEK and ERK. ERK, in turn, phosphorylates and dis‐inhibits Caldesmon (CaD) to remove an inhibitory action on the contractile filament leading to an activation of SMC and increased stiffness.^27^, ^59^ Our present results show that NMII increases phosphorylation of FA proteins that cause FA growth and generate cortical tension to increase cellular stiffness.^27^ Additionally, the cortical activation of ERK leads to targeting of activated ERK to the contractile filaments and disinhibition of CaD, which further increases aortic contractility and stiffness (Figure 7). Thus, the inhibition of NMII by blebbistatin results in less cytoskeletal tension, which prevents phosphorylation of FAK and paxillin and ultimately leads to decreased stiffness and stress (Figure 7). Taken together, it appears that NMII regulates aortic stiffness by inhibiting cross‐bridge cycling and also by decreasing tension in the non‐muscle actin cytoskeleton through prevention of phosphorylation of FA proteins.

## CONFLICT OF INTEREST

The authors confirm that there are no conflicts of interest.

## AUTHOR CONTRIBUTIONS

**Kuldeep Singh:** Conceptualization (equal); Data curation (lead); Formal analysis (lead); Investigation (lead); Methodology (lead); Supervision (supporting); Validation (equal); Visualization (lead); Writing‐original draft (lead); Writing‐review & editing (equal). **Anne B Kim:** Data curation (supporting); Formal analysis (supporting); Investigation (supporting); Methodology (supporting). **Kathleen G. Morgan:** Conceptualization (equal); Funding acquisition (lead); Investigation (supporting); Methodology (supporting); Project administration (lead); Validation (equal); Visualization (supporting); Writing‐original draft (supporting); Writing‐review & editing (equal).

## Data Availability

The data that support the finding of this study are available from the corresponding author upon reasonable request.
